# Sato-Tate distribution of *p*-adic hypergeometric functions

**DOI:** 10.1007/s40993-022-00414-w

**Published:** 2022-11-29

**Authors:** Sudhir Pujahari, Neelam Saikia

**Affiliations:** 1 School of Mathematical Sciences, National Institute of Science Education and Research,Bhubaneswar, An OCC of Homi Bhabha National Institute, P. O. Jatni, Khurda, Odisha 752050 India; 2 Faculty of Mathematics, University of Vienna, Vienna, 1010 Austria

**Keywords:** *p*-adic hypergeometric functions, Sato-Tate distributions, Trace of Frobenius, 11G20, 11T24, 33E50

## Abstract

Recently Ono, Saad and the second author [21] initiated a study of value distribution of certain families of Gaussian hypergeometric functions over large finite fields. They investigated two families of Gaussian hypergeometric functions and showed that they satisfy semicircular and Batman distributions. Motivated by their results we aim to study distributions of certain families of hypergeometric functions in the *p*-adic setting over large finite fields. In particular, we consider two and six parameters families of hypergeometric functions in the *p*-adic setting and obtain that their limiting distributions are semicircular over large finite fields. In the process of doing this we also express the traces of *p*th Hecke operators acting on the spaces of cusp forms of even weight $k\ge 4$ and levels 4 and 8 in terms of *p*-adic hypergeometric function which is of independent interest. These results can be viewed as *p*-adic analogous of some trace formulas of [1, 2, 6].

## Introduction

Let *p* be an odd prime and let $\mathbb {F}_q$ denote the finite field with $q=p^r$ elements. In [11], Greene introduced Gaussian hypergeometric functions over finite fields using Jacobi sums. An important fact regarding these functions is that they satisfy many identities that are analogous to those satisfied by classical hypergeometric functions. Recently Ono, Saad and the second author [21] initiated a study of value distributions of certain families of these functions over random large finite fields $\mathbb {F}_q.$ More precisely, they investigated the distributions of the normalized values of the following two Gaussian hypergeometric functions over $\mathbb {F}_q$ that are defined by

$$\begin{aligned} _2F_1(\lambda )_q:={_2F_1}\left( \begin{matrix} \phi , &{} \phi \\ ~ &{}\varepsilon \end{matrix}\mid \lambda \right) _q: =\frac{q}{q-1}\sum \limits _{\chi }{\phi \chi \atopwithdelims ()\chi } {\phi \chi \atopwithdelims ()\chi }\chi (\lambda ) \end{aligned}$$

and

$$\begin{aligned} _3F_2(\lambda )_q:={_3F_2}\left( \begin{matrix} \phi , &{} \phi , &{} \phi \\ ~ &{}\varepsilon , &{} \varepsilon \end{matrix}\mid \lambda \right) _q: =\frac{q}{q-1}\sum \limits _{\chi }{\phi \chi \atopwithdelims ()\chi }{\phi \chi \atopwithdelims ()\chi }{\phi \chi \atopwithdelims ()\chi }\chi (\lambda ), \end{aligned}$$

where $\phi$ and $\varepsilon$ are quadratic and trivial characters of $\mathbb {F}_q^{\times }$ and the sums on the far rights run over all multiplicative characters^1^ of $\mathbb {F}_q.$ Moreover, the symbol ${A\atopwithdelims ()B}:=\frac{B(-1)}{q}J(A,\overline{B}):=\frac{B(-1)}{q}\sum \limits _{x\in \mathbb {F}_q}A(x)\overline{B}(1-x)$ is the normalized Jacobi sum. Ono et al. [21] showed that for the $_2F_1(\lambda )_q$ functions the limiting distribution is semicircular whereas the distribution for the $_3F_2(\lambda )_q$ functions is the Batman distribution for the traces of the real orthogonal group *O*(3). In this paper our goal is to study similar questions for *p*-adic hypergeometric functions over random large finite fields.

McCarthy [17, 18] defined *p*-adic hypergeometric functions using *p*-adic gamma functions extending Greene’s hypergeometric functions [11] for wider classes of primes. These functions appear in the study of Frobenius trace of elliptic curves [17], Fourier coefficients of Hecke eigen forms [22], and proofs of supercongruence type identities [10].

Let $\Gamma _p(\cdot )$ be the Morita’s *p*-adic gamma function (see [16]). Let $\omega$ be the Teichmüller character^2^ of $\mathbb {F}_p$ and $\overline{\omega }$ denote its character inverse. For $x\in \mathbb {Q}$ let $\lfloor x\rfloor$ denote the greatest integer less than or equal to *x* and $\langle x\rangle$ denote the fractional part of *x*, satisfying $0\le \langle x\rangle <1$. In these notation *p*-adic hypergeometric function is defined as follows.

### Definition 1.1

[17, Definition 1.1] Let *p* be an odd prime and $t \in \mathbb {F}_p$. For positive integer *n* and $1\le k\le n$, let $a_k$, $b_k$ $\in \mathbb {Q}\cap \mathbb {Z}_p$. Then

$$\begin{aligned}&_n{G}_n\left[ \begin{array}{cccc} a_1, &{} a_2, &{} \ldots , &{} a_n \\ b_1, &{} b_2, &{} \ldots , &{} b_n \end{array}\mid t \right] _p:=\frac{-1}{p-1}\sum _{j=0}^{p-2}(-1)^{jn}~~\overline{\omega }^j(t)\nonumber \nonumber \\&\times \prod \limits _{k=1}^n(-p)^{-\lfloor \langle a_k \rangle -\frac{j}{p-1} \rfloor -\lfloor \langle -b_k \rangle +\frac{j}{p-1}\rfloor } \frac{\Gamma _p(\langle a_k-\frac{j}{p-1}\rangle )}{\Gamma _p(\langle a_k \rangle )} \frac{\Gamma _p(\langle -b_k+\frac{j}{p-1} \rangle )}{\Gamma _p(\langle -b_k \rangle )}.\nonumber \end{aligned}$$

In this paper we aim to investigate the value distributions of certain families of these functions over large finite fields $\mathbb {F}_p$. Namely, we study the value distributions of the following families of $_nG_n$-functions for $n=2,6.$ Let $\psi _6=\omega ^{\frac{p-1}{6}}$ and $\psi _3=\omega ^{\frac{p-1}{3}}$ be characters of order 6 and 3, respectively. Then for $\lambda \in \mathbb {F}_p$ we define

$$\begin{aligned} _2G_2(\lambda )_p:=p \psi _6(2)\phi (1+\lambda )\overline{\psi }_3(2\lambda (1+\lambda ))\cdot {_2G_2}\left[ \begin{array}{cc} \frac{2}{3}, &{} \frac{2}{3} \\ \frac{5}{12}, &{} \frac{11}{12} \end{array}\mid \frac{4\lambda }{(1+\lambda )^2} \right] _p. \end{aligned}$$

and

$$\begin{aligned} _6G_6(\lambda )_p:=\phi (1+\lambda )\cdot {_6G_6}\left[ \begin{array}{cccccc} \frac{1}{3}, &{} \frac{1}{3}, &{} \frac{2}{3}, &{} \frac{2}{3}, &{} 0, &{} 0 \\ \frac{1}{12}, &{} \frac{1}{4}, &{} \frac{5}{12}, &{} \frac{7}{12}, &{} \frac{3}{4}, &{} \frac{11}{12} \end{array}\mid \frac{2^6\lambda ^3}{(1+\lambda )^6} \right] _p. \end{aligned}$$

As our first theorem we obtain the moments of values of ${_2G_2}(\lambda )_p.$

### Theorem 1.2

Let *m* be a fixed positive integer and $p\equiv 1\pmod {3}$ be a prime. Then as $p\rightarrow \infty$

$$\begin{aligned} \sum \limits _{\lambda \in \mathbb {F}_p}{_2G_2}(\lambda )_p^{m}= {\left\{ \begin{array}{ll} o_{m}(p^{\frac{m}{2}+1}) &{} \text {if}\ \ m \ is\ \text {odd} \\ \frac{(2n)!}{n!(n+1)!}p^{n+1}+o_m(p^{n+1}) &{} \text {if}\ \ m=2n \ is\ \text {even}. \end{array}\right. } \end{aligned}$$

We use these moments to conclude the limiting behaviour of ${_2G_2(\lambda )_p}$ as $p\rightarrow \infty .$ If we view the normalized values $p^{-1/2}\cdot {_2G_2(\lambda )_p}\in [-2,2]$ as random variables over $\mathbb {F}_p$ then we obtain the limiting distribution of this function. More precisely we have the following distribution.

### Corollary 1.3

If $$-2\le a<b\le 2$$ and $$p\equiv 1\pmod {3}$$

$$\begin{aligned} \lim \limits _{p\rightarrow \infty }\frac{|\{\lambda \in \mathbb {F}_p:\ \ p^{-1/2} {_2G_2}(\lambda )_p\in [a,b]\}|}{p}=\frac{1}{2\pi }\int _a^b\sqrt{4-t^2}~dt. \end{aligned}$$

We also consider these problems for the ${_6G_6(\lambda )_p}$ functions.

### Theorem 1.4

Let *m* be a fixed positive integer and $p\equiv 2\pmod {3}$ be a prime. Then as $p\rightarrow \infty$

$$\begin{aligned} \sum \limits _{\lambda \in \mathbb {F}_p}{_6G_6}(\lambda )_p^{m}= {\left\{ \begin{array}{ll} o_{m}(p^{\frac{m}{2}+1}) &{} \text {if}\ \ m \ is\ \text {odd} \\ \frac{(2n)!}{n!(n+1)!}p^{n+1}+o_m(p^{n+1}) &{} \text {if}\ \ m=2n \ is\ \text {even}. \end{array}\right. } \end{aligned}$$

Similarly as in Corollary 1.3, we conclude the limiting distribution of $\frac{{_6G_6}(\lambda )_p}{\sqrt{p}}\in [-2,2]$ in the following Corollary.

### Corollary 1.5

If $-2\le a<b\le 2$ and $p\equiv 2\pmod {3}$ then

$$\begin{aligned} \lim \limits _{p\rightarrow \infty }\frac{|\{\lambda \in \mathbb {F}_p:\ \ p^{-1/2}\cdot {_6G_6}(\lambda )_p\in [a,b]\}|}{p}=\frac{1}{2\pi }\int _a^b \sqrt{4-t^2}~dt. \end{aligned}$$

### Remark 1.6

It is important to note that these results can be extended to $_2G_2(\lambda )_q$ and $_6G_6(\lambda )_q$ over finite fields $\mathbb {F}_q$ where $q=p^r$ using similar arguments. For simplicity we choose $q=p.$

## Traces of Hecke operators and hypergeometric functions

It turns out that the hypergeometric functions $_2G_2(\lambda )_p$ and $_6G_6(\lambda )_p$ can be related to the traces of Hecke operators acting on the spaces of cusp forms. More precisely, for positive integers *N* and *k*, let *S*(*N*, *k*) be the space of cusp forms of weight *k* with respect to the congruence subgroup $\Gamma _0(N)$ and let ${\textrm{Tr}_k}(\Gamma _0(N),p)$ denote the trace of the *p*th Hecke operator acting on the space *S*(*N*, *k*). In a series of papers Ahlgren [1], Ahlgren-Ono [2] and Frechette-Ono-Papanikolas [6] studied the Eichler-Selberg type trace formulas for the *p*th Hecke operators acting on the spaces *S*(*N*, *k*) for $N=2,4,8$ and established trace formulas using Gaussian hypergeometric functions over finite fields. Here we obtain the following results expressing the trace formulas ${\textrm{Tr}_k}(\Gamma _0(4),p)$ and ${\textrm{Tr}_k}(\Gamma _0(8),p)$ using *p*-adic hypergeometric functions $_2G_2(\lambda )_p$ and $_6G_6(\lambda )_p$. Note that both these functions vanish at $\lambda =-1,$ therefore, we define refinements of these functions. To this end we define the following two functions.

$$\begin{aligned} {_2\widetilde{G}_2}(\lambda )_p:= {_2G_2}(\lambda )_p-\delta (1+\lambda )\cdot \Delta (p)\cdot A_4(-1)\textrm{Re}\left( \frac{g(\phi A_4)g(\overline{A}_4)}{g(\phi )}\right) \end{aligned}$$

and

$$\begin{aligned} {_6\widetilde{G}_6}(\lambda )_p:= {_6G_6}(\lambda )_p-\delta (1+\lambda )\cdot \Delta (p)\cdot A_4(-1)\textrm{Re}\left( \frac{g(\phi A_4)g(\overline{A}_4)}{g(\phi )}\right) , \end{aligned}$$

where $A_4$ is a character of order 4,$$\delta (x):={\left\{ \begin{array}{ll} 1, &{} \text {if} \ x=0\\ 0, &{} \text {if} \ x\ne 0, \end{array}\right. } $$ and $$\Delta (p):={\left\{ \begin{array}{ll} 1, &{} \text {if} \ p\equiv 1\pmod {4}\\ 0, &{} \text {if} \ p\equiv 3\pmod {4}. \end{array}\right. }$$ Moreover, let $P_k(s,p)$ be a polynomial defined as follows

$$\begin{aligned} P_k(s,p):=\sum \limits _{j=0}^{k/2-1}(-1)^j{k-2-j \atopwithdelims ()j}p^j s^{k-2j-2}. \end{aligned}$$

In this notation, we have the following results.

### Theorem 2.1

If $p\equiv 1\pmod 3$ is an odd prime and $k\ge 4$ is even then

$$\begin{aligned} {\textrm{Tr}_k}(\Gamma _0(4),p)=-3-\sum _{\lambda =2}^{p-1}P_k(_2\widetilde{G}_2(\lambda )_p,p) \end{aligned}$$

and

$$\begin{aligned} {\textrm{Tr}_k}(\Gamma _0(8),p)=-4-\sum _{\lambda =2}^{p-1} P_k(_2\widetilde{G}_2(\lambda ^2)_p,p). \end{aligned}$$

### Theorem 2.2

If $p\equiv 2\pmod 3$ is an odd prime and $k\ge 4$ is even then

$$\begin{aligned} {\textrm{Tr}_k}(\Gamma _0(4),p)=-3-\sum _{\lambda =2}^{p-1}P_k(_6\widetilde{G}_6 (\lambda )_p,p) \end{aligned}$$

and

$$\begin{aligned} {\textrm{Tr}_k}(\Gamma _0(8),p)=-4-\sum _{x=2}^{p-1}P_k(_6\widetilde{G}_6(\lambda ^2)_p,p). \end{aligned}$$

The paper is organized as follows. In Sect. 3 we provide some important results on *p*-adic gamma functions and Gauss sums. Namely, the Davenport-Hasse Relation and the Gross-Koblitz formula. Sect. 4 contains the proof of the main results.

## Gauss sums and *p*-adic gamma function

In this section we discuss some important theorems, namely the Gross-Koblitz formula and Davenport-Hasse relation. We also recall some basic results regarding Gauss sums and *p*-adic gamma functions. We begin by recalling the orthogonality relations satisfied by multiplicative characters. Let $\widehat{\mathbb {F}_p^\times }$ denote the cyclic group of multiplicative characters of $\mathbb {F}_p^{\times }.$

### Lemma 3.1

([13, Chapter 8]). If $\chi$ is a multiplicative character^3^ of $\mathbb {F}_p^\times ,$ then (1) $$\sum \limits _{\chi \in \widehat{\mathbb {F}_p^\times }}\chi (x)~~=\left\{ \begin{array}{ll} p-1 &{} \hbox {if}~~ x=1; \\ 0 &{} \hbox {if}~~ x\ne 1. \end{array} \right. $$

(2) $$\sum \limits _{x\in \mathbb {F}_p}\chi (x)~~=\left\{ \begin{array}{ll} p-1 &{} \hbox {if}~~ \chi =\varepsilon ; \\ 0 &{} \hbox {if} ~~\chi \ne \varepsilon . \end{array} \right. $$

Let $\zeta _p$ be a primitive *p*th root of unity and $\chi$ be a multiplicative character ${\mathbb {F}_p^\times }.$ Then the Gauss sum is defined by

$$\begin{aligned} g(\chi ):=\sum \limits _{x\in \mathbb {F}_p}\chi (x)\zeta _p^{x}. \end{aligned}$$

### Theorem 3.2

([3, Davenport-Hasse Relation]). Let *n* be a positive integer and let *p* be a prime such that $p\equiv 1 \pmod {n}$. For multiplicative characters $\chi , \psi \in \widehat{\mathbb {F}_p^\times }$, we have

$$\begin{aligned} \prod \limits _{\chi ^n=\varepsilon }g(\chi \psi )=-g(\psi ^n)\psi (n^{-n})\prod \limits _{\chi ^n=\varepsilon }g(\chi ).\nonumber \end{aligned}$$

Let $\mathbb {Z}_p$ and $\mathbb {Q}_p$ denote the ring of *p*-adic integers and the field of *p*-adic numbers, respectively. Let $\overline{\mathbb {Q}_p}$ be the algebraic closure of $\mathbb {Q}_p$ and $\mathbb {C}_p$ be the completion of $\overline{\mathbb {Q}_p}$. For a positive integer *n* the *p*-adic gamma function $\Gamma _p(n)$ is defined as

$$\begin{aligned} \Gamma _p(n):=(-1)^n\prod \limits _{\begin{array}{c} 0<j<n \\ p\not \mid j \end{array}}j.\nonumber \end{aligned}$$

The domain of definition of the p-adic gamma function can be extended to all $x\in \mathbb {Z}_p$ by simply setting $\Gamma _p(0):=1$ and for $x\ne 0$

$$\begin{aligned} \Gamma _p(x):=\lim _{x_n\rightarrow x}\Gamma _p(x_n),\nonumber \end{aligned}$$

where $x_n$ is any sequence of positive integers *p*-adically approaching to *x*. For more details on *p*-adic gamma functions we refer [16]. To this end we recall Gross-Koblitz formula which relates Gauss sums to *p*-adic gamma functions. Let $\pi \in \mathbb {C}_p$ be the fixed root of the polynomial $x^{p-1} + p$, which satisfies the congruence condition $\pi \equiv \zeta _p-1 \pmod {(\zeta _p-1)^2}$.

### Theorem 3.3

[12, Gross-Koblitz]. For $a\in \mathbb {Z}$,

$$\begin{aligned} g(\overline{\omega }^a)=-\pi ^{(p-1)\langle \frac{a}{p-1} \rangle }\Gamma _p\left( \left\langle \frac{a}{p-1} \right\rangle \right) .\nonumber \end{aligned}$$

Now we recall (see [16]) a *p*-adic analogue of the Davenport-Hasse relation and a product formula of *p*-adic gamma functions. If $n\in \mathbb {Z}^{+}$, $p\not \mid n$ and $x=\frac{r}{p-1}$ with $0\le r\le p-1$, then

3.1 $$\begin{aligned} \prod _{h=0}^{n-1}\Gamma _p\left( \frac{x+h}{n}\right) =\omega (n^{(1-x)(1-p)}) \Gamma _p(x)\prod _{h=1}^{n-1}\Gamma _p\left( \frac{h}{n}\right) \end{aligned}$$

and we also note that

3.2 $$\begin{aligned} \Gamma _p(x)\Gamma _p(1-x)=(-1)^{x_0}, \end{aligned}$$

where $$x_0\in \{1,2,\ldots , p\}$$ satisfies $$x\equiv x_0\pmod {p}.$$

### Lemma 3.4

[17, Lemma 4.1]. Let *p* be a prime and $0\le j\le p-2$. For $n\ge 1$ with $p\not \mid n$, we have

$$\begin{aligned} \omega (n^{nj})\Gamma _p\left( \left\langle \frac{nj}{p-1}\right\rangle \right) \prod \limits _{h=1}^{n-1}\Gamma _p\left( \left\langle \frac{h}{n}\right\rangle \right) =\prod \limits _{h=0}^{n-1}\Gamma _p\left( \left\langle \frac{h}{n}+\frac{j}{p-1}\right\rangle \right) ,\nonumber \end{aligned}$$

and

$$\begin{aligned} \omega (n^{-nj})\Gamma _p\left( \left\langle \frac{-nj}{p-1}\right\rangle \right) \prod \limits _{h=1}^{n-1}\Gamma _p\left( \left\langle \frac{h}{n}\right\rangle \right) =\prod \limits _{h=0}^{n-1}\Gamma _p\left( \left\langle \frac{1+h}{n}-\frac{j}{p-1}\right\rangle \right) .\nonumber \end{aligned}$$

## Proofs of theorems

For $\lambda \ne 0,1,$ let $E_{\lambda }^{\textrm{Leg}}:\ \ y^2=x(x-1)(x-\lambda )$ be the Legendre normal form of elliptic curve over $\mathbb {F}_p$ and

$$\begin{aligned} a_p(\lambda ):=p+1-|E_{\lambda }^{\textrm{Leg}}(\mathbb {F}_p)|=-\sum \limits _{x\in \mathbb {F}_p}{\phi (x(x-1)(x-\lambda ))} \end{aligned}$$

be the trace of Frobenius of the elliptic curve $E_{\lambda }^{\textrm{Leg}}.$ Our main idea is to express the functions $_2G_2(\lambda )_p$ and $_6G_6(\lambda )_p$ in terms of the traces of Frobenius $a_p(\lambda ).$ Therefore, to obtain the asymptotic formulas of power moments of these functions it is sufficient if we have asymptotic formulas for the power moments of $a_p(\lambda ).$ In [21] Ono et al. computed the asymptotic formulas for power moments of the functions $_2F_1(\lambda )_p.$ Here we reformulate their formulas in terms of power moments of $a_p(\lambda )$ in the following theorem.

### Theorem 4.1

Let *m* be a fixed positive integer and $p>3$ be a prime. Then as $p\rightarrow \infty$

4.1 $$\begin{aligned} \sum \limits _{\lambda \ne 0,1}a_p(\lambda )^m= {\left\{ \begin{array}{ll} o_{m}(p^{\frac{m}{2}+1}) &{} \text {if}\ \ m \ is\ \text {odd} \\ \frac{(2n)!}{n!(n+1)!}p^{n+1}+o_m(p^{n+1}) &{} \text {if}\ \ m=2n \ is\ \text {even}. \end{array}\right. } \end{aligned}$$

### Proof

The proof follows easily by making use of Theorem 1 of [20], Theorem 1.1 of [21] and the value $_2F_1(1)_p=-\phi (-1)/p.$ $\square$

Theorem 4.1 is a refinement of a classical theorem of Birch [4, Theorem 1] (when restricted to Legendre normal elliptic curves) that established even moments of all elliptic curves over finite fields. Recently Bringmann, Kane, and the first author [5, 14] have refined Birch’s result to arithmetic progressions.

### Proof of Theorem 1.2

By the definition we can write

$$\begin{aligned} {_2G_2}(\lambda )_p&=\frac{p\cdot \psi _6(2)\phi (1+\lambda )\overline{\psi }_3(2\lambda (1+\lambda ))}{(1-p)\Gamma _p(\frac{1}{12})\Gamma _p(\frac{7}{12}) \Gamma _p(\frac{2}{3})^2} \sum _{j=0}^{p-2}\overline{\omega }^j\left( \frac{4\lambda }{(1+\lambda )^2}\right) \nonumber \\&\quad \times (-p)^{-\left\lfloor \frac{1}{12}+\frac{j}{p-1} \right\rfloor -\left\lfloor \frac{7}{12}+\frac{j}{p-1}\right\rfloor -2\left\lfloor \frac{2}{3}-\frac{j}{p-1}\right\rfloor }~ \Gamma _p\left( \left\langle \frac{1}{12}+\frac{j}{p-1} \right\rangle \right) \Gamma _p\left( \left\langle \frac{7}{12}+\frac{j}{p-1} \right\rangle \right) \nonumber \\&\quad \times \Gamma _p\left( \left\langle \frac{2}{3}-\frac{j}{p-1} \right\rangle \right) ^2.\nonumber \end{aligned}$$

Choosing $x=\frac{1}{6}$ and $n=2$ in (3.1) we obtain

$$\begin{aligned} \Gamma _p\left( \frac{1}{12}\right) \Gamma _p\left( \frac{7}{12}\right) =\overline{\omega }^{\frac{5(p-1)}{6}}(2)\Gamma _p\left( \frac{1}{6}\right) \Gamma _p\left( \frac{1}{2}\right) . \end{aligned}$$

Again, choosing $x=\frac{1}{3}$ and $n=2$ in (3.1) we have

$$\begin{aligned} \Gamma _p\left( \frac{1}{6}\right) \Gamma _p\left( \frac{2}{3}\right) =\overline{\omega }^{\frac{2(p-1)}{3}}(2)\Gamma _p\left( \frac{1}{3}\right) \Gamma _p\left( \frac{1}{2}\right) . \end{aligned}$$

Using these two expressions we obtain $\Gamma _p(\frac{1}{12})\Gamma _p(\frac{7}{12}) \Gamma _p(\frac{2}{3})^2=\phi (2)\Gamma _p(\frac{1}{3})\Gamma _p(\frac{2}{3})\Gamma _p(\frac{1}{2})^2.$ Moreover, if we use product formula (3.2) twice with $x=\frac{2}{3}$ and $\frac{1}{2}$ then we have $\Gamma _p(\frac{1}{3})\Gamma _p(\frac{2}{3})=-1$ and $\Gamma _p(\frac{1}{2})^2=-\phi (-1).$ These give $\Gamma _p(\frac{1}{12})\Gamma _p(\frac{7}{12}) \Gamma _p(\frac{2}{3})^2=\phi (-2).$ Now, substituting this value and taking the transformation $j\rightarrow j+\frac{p-1}{3}$ we have

4.2 $$\begin{aligned} {_2G_2}(\lambda )_p&=\frac{p\cdot \phi (-2)\psi _6(2) \phi (1+\lambda )}{1-p}{\psi }_3(\lambda (1+\lambda )) \sum _{j=0}^{p-2}\overline{\omega }^j\left( \frac{4\lambda }{(1+\lambda )^2}\right) \nonumber \\&\quad \times (-p)^{-\left\lfloor \frac{5}{12}+\frac{j}{p-1} \right\rfloor -\left\lfloor \frac{11}{12}+\frac{j}{p-1}\right\rfloor -2\left\lfloor \frac{1}{3}-\frac{j}{p-1}\right\rfloor }~ \Gamma _p\left( \left\langle \frac{5}{12}+\frac{j}{p-1} \right\rangle \right) \Gamma _p\left( \left\langle \frac{11}{12}+\frac{j}{p-1} \right\rangle \right) \nonumber \\&\quad \times \Gamma _p\left( \left\langle \frac{1}{3}-\frac{j}{p-1} \right\rangle \right) ^2. \end{aligned}$$

If we put $n=2$ and $x=\langle \frac{5}{6}+\frac{2j}{p-1}\rangle$ in (3.1), then we have

$$\begin{aligned} \Gamma _p\left( \left\langle \frac{5}{6}+\frac{2j}{p-1}\right\rangle \right) = \overline{\omega }^{j}(4)\psi _6(2) \frac{\Gamma _p(\langle \frac{5}{12}+\frac{j}{p-1}\rangle )\Gamma _p(\langle \frac{11}{12}+\frac{j}{p-1}\rangle )}{\Gamma _p(\frac{1}{2})}. \end{aligned}$$

Also it is easy to verify that

$$\begin{aligned} \left\lfloor \frac{5}{6}+\frac{2j}{p-1}\right\rfloor =\left\lfloor \frac{5}{12}+\frac{j}{p-1}\right\rfloor +\left\lfloor \frac{11}{12}+\frac{j}{p-1}\right\rfloor . \end{aligned}$$

Using these two facts in (4.2) we obtain

4.3 $$\begin{aligned} {_2G_2}(\lambda )_p&=\frac{p\Gamma _p(\frac{1}{2})\phi (1+\lambda )}{1-p}\phi (-2) {\psi }_3(\lambda (1+\lambda )) \sum _{j=0}^{p-2}\overline{\omega }^j\left( \frac{\lambda }{(1+\lambda )^2}\right) \nonumber \\&\times (-p)^{-\left\lfloor \frac{5}{6}+\frac{2j}{p-1}\right\rfloor -2\left\lfloor \frac{1}{3}-\frac{j}{p-1}\right\rfloor }~ \Gamma _p\left( \left\langle \frac{5}{6}+\frac{2j}{p-1} \right\rangle \right) \Gamma _p\left( \left\langle \frac{1}{3}-\frac{j}{p-1} \right\rangle \right) ^2. \end{aligned}$$

Now, Gross-Koblitz formula allow us to write

$$\begin{aligned} {_2G_2}(\lambda )_p=\frac{\phi (2)\phi (1+\lambda )}{(1-p)} {\psi }_3(\lambda (1+\lambda )) \sum _{j=0}^{p-2}\overline{\omega }^j\left( \frac{\lambda }{(1+\lambda )^2}\right) \frac{g(\overline{\psi }_6^5\overline{\omega }^{2j})g(\overline{\psi }_3\omega ^j)^2}{g(\phi )}. \end{aligned}$$

Replacing $\overline{\omega }^j$ by $\overline{\omega }^j\overline{\psi }_3$ we obtain

4.4 $$\begin{aligned} {_2G_2}(\lambda )_p&=\frac{\phi (2)\phi (1+\lambda )}{(1-p)} \sum _{j=0}^{p-2}\overline{\omega }^j\left( \frac{\lambda }{(1+\lambda )^2}\right) \frac{g(\phi \overline{\omega }^{2j})g(\omega ^j)^2}{g(\phi )}\nonumber \\&=\frac{\phi (2)\phi (1+\lambda )}{(1-p)g(\phi )}\sum _{u,v,y\in \mathbb {F}_p}\phi (u)\zeta _p^{u+v+y}\sum _{j=0}^{p-2}\omega ^j\left( \frac{yv(1+\lambda )^2}{u^2\lambda }\right) . \end{aligned}$$

Using the orthogonality of multiplicative characters and then replacing *u* by $-(1+\lambda )uy$ we deduce that

$$\begin{aligned} {_2G_2}(\lambda )_p=-\phi (-2)\sum \limits _{u\in \mathbb {F}_p}\phi (u(1-u)(1-\lambda u)). \end{aligned}$$

Again transforming *u* by 1/*u* we obtain

4.5 $$\begin{aligned} {_2G_2}(\lambda )_p=-\phi (-2)\sum \limits _{u\in \mathbb {F}_p}\phi (u(u-1) (u-\lambda )). \end{aligned}$$

Therefore, for $\lambda \ne 0,\pm 1$ we have

4.6 $$\begin{aligned} _2G_2(\lambda )_p=\phi (-2)a_p(\lambda ). \end{aligned}$$

Using this relation we write

4.7 $$\begin{aligned} \sum \limits _{\lambda \in \mathbb {F}_p} {_2G_2}(\lambda )_p^m= {_2G_2}(1)_p^m -\phi (-2)^m a_p(-1)^m +\phi (-2)^m\sum \limits _{\lambda \ne 0,1} a_p(\lambda )^m. \end{aligned}$$

By (4.5) and second part of Lemma 3.1 it is easy to see that

4.8 $$\begin{aligned} {_2G_2}(1)_p=\phi (-2). \end{aligned}$$

By Hasse bound we have

4.9 $$\begin{aligned} a_p(-1)^m=o(p^{m/2+1}). \end{aligned}$$

Finally, applying Theorem 4.1, (4.8) and (4.9) in (4.7) we conclude the result.

$$\square $$

### Proof of Theorem 1.4

Let

$$\begin{aligned} H(\lambda )={_6G_6}\left[ \begin{array}{cccccc} \frac{1}{12}, &{} \frac{1}{4}, &{} \frac{5}{12}, &{} \frac{7}{12}, &{} \frac{3}{4}, &{} \frac{11}{12} \\ \frac{1}{3}, &{} \frac{1}{3}, &{} \frac{2}{3}, &{} \frac{2}{3}, &{} 0, &{} 0 \end{array}\mid \frac{(1+\lambda )^6}{2^6\lambda ^3} \right] _p. \end{aligned}$$

By definition, we write

4.10 $$\begin{aligned} H(\lambda )&=\frac{1}{1-p}\sum _{j=0}^{p-2}{\omega }^{j}\left( \frac{2^6\lambda ^3}{(1+\lambda )^6}\right) \pi ^{(p-1)S_j}\nonumber \\&\times \prod _{\begin{array}{c} h=1\\ ~h~odd \end{array}}^{11}\frac{\Gamma _p(\langle \frac{h}{12}- \frac{j}{p-1}\rangle )}{\Gamma _p(\langle \frac{h}{12}\rangle )}\times \frac{\Gamma _p(\langle \frac{1}{3}+\frac{j}{p-1}\rangle )^2~\Gamma _p(\langle \frac{2}{3}+\frac{j}{p-1}\rangle )^2~\Gamma _p(\langle \frac{j}{p-1}\rangle )^2}{\Gamma _p(\langle \frac{1}{3}\rangle )^2~\Gamma _p(\langle \frac{2}{3}\rangle )^2}, \end{aligned}$$

where

$$\begin{aligned} S_j=-2\left\lfloor \frac{j}{p-1}\right\rfloor -2\left\lfloor \frac{1}{3}+\frac{j}{p-1}\right\rfloor -2\left\lfloor \frac{2}{3}+\frac{j}{p-1}\right\rfloor -\sum \limits _{\begin{array}{c} h=1\\ h~odd \end{array}}^{12}\left\lfloor \frac{h}{12}-\frac{j}{p-1}\right\rfloor . \end{aligned}$$

Now applying Lemma 3.4 we deduce that

$$\begin{aligned} H(\lambda )=\frac{1}{1-p}\sum \limits _{j=0}^{p-2}{\omega }^{j} \left( \frac{\lambda ^3}{2^{12}(1+\lambda )^6}\right) \pi ^{(p-1)S_j} \frac{\Gamma _p(\langle \frac{-12j}{p-1}\rangle )\left( \Gamma _p(\langle \frac{3j}{p-1}\rangle )\right) ^2}{\Gamma _p(\langle \frac{-6j}{p-1}\rangle )}. \end{aligned}$$

For $1\le j\le p-2$ it is easy to verify that $\left\lfloor \frac{-12j}{p-1}\right\rfloor =\sum \limits _{h=0}^{11}\left\lfloor \frac{h}{12}-\frac{j}{p-1}\right\rfloor$ and

$$ \left\lfloor \frac{-6j}{p-1}\right\rfloor =\sum \limits _{h=0}^{5}\left\lfloor \frac{h}{6}-\frac{j}{p-1}\right\rfloor . $$ Moreover, $$ \left\lfloor \frac{3j}{p-1}\right\rfloor = \left\lfloor \frac{j}{p-1}\right\rfloor +\left\lfloor \frac{1}{3}+\frac{j}{p-1}\right\rfloor +\left\lfloor \frac{2}{3}+\frac{j}{p-1}\right\rfloor . $$

If we use these three relations in the exponent of $\pi$ present in $H(\lambda )$ then the Gross-Koblitz formula allows to write

$$\begin{aligned} H(\lambda )=\frac{1}{(1-p)}\sum _{j=0}^{p-2} \frac{g({\omega }^{12j})g(\overline{\omega }^{3j})^2}{g({\omega }^{6j})} {\omega }^{j}\left( \frac{\lambda ^3}{2^{12}(1+\lambda )^6}\right) . \end{aligned}$$

By making use of Davenport-Hasse relation one can have

4.11 $$\begin{aligned} g({\omega }^{6j})g(\phi {\omega }^{6j}) =g({\omega }^{12j}){\omega }^{6j}(2^{-2})g(\phi ). \end{aligned}$$

Therefore, using (4.11) we have

$$\begin{aligned} H(\lambda )=\frac{1}{(1-p)}\sum _{j=0}^{p-2} \frac{g(\phi {\omega }^{6j})g(\overline{\omega }^{3j})^2}{g(\phi )} {\omega }^{j}\left( \frac{\lambda ^3}{(1+\lambda )^6}\right) . \end{aligned}$$

Since $p\equiv 2\pmod {3}$ therefore transforming $j\rightarrow -j/3$ we obtain

$$\begin{aligned} H(\lambda )=\frac{1}{(1-p)}\sum _{j=0}^{p-2} \frac{g(\phi \overline{\omega }^{2j})g(\omega ^{j})^2}{g(\phi )} \overline{\omega }^{j}\left( \frac{\lambda }{(1+\lambda )^2}\right) . \end{aligned}$$

Now observe that the above sum is equal to the sum involved in (4.4) up to a scalar multiple. Therefore following similar steps we obtain

$$\begin{aligned} \phi (1+\lambda )H(\lambda )=\phi (-1)\cdot a_p(\lambda ). \end{aligned}$$

Therefore, if $\lambda \ne 0,\pm 1$ then

4.12 $$\begin{aligned} _6G_6(\lambda )_p=\phi (-1)\cdot a_p(\lambda ). \end{aligned}$$

The rest of the proof relies on similar arguments as in the proof of Theorem 1.2.

$$\square $$

### Proof of Theorem 2.1

If $\lambda \ne 0,\pm 1,$ then (4.6) gives $_2\widetilde{G}_2(\lambda )_p=a_p(\lambda ).$

Using [11, eqn (2.10)] and the orthogonality relation Lemma 3.1 one can verify that

4.13 $$\begin{aligned} a_p(-1)&={\left\{ \begin{array}{ll} 0, &{} \text {if}\ p\equiv 3\pmod {4}\\ -p\left( {\phi A_4\atopwithdelims ()A_4}+{\phi \overline{A}_4\atopwithdelims ()\overline{A}_4}\right) , &{} \text {if}\ p\equiv 1\pmod {4}, \end{array}\right. }\nonumber \\&= {\left\{ \begin{array}{ll} 0, &{} \text {if}\ p\equiv 3\pmod {4}\\ - A_4(-1)\left( \frac{g(\phi A_4)g(\overline{A}_4)}{g(\phi )} +\frac{g(\phi \overline{A}_4)g(A_4)}{g(\phi )}\right) , &{} \text {if}\ p\equiv 1\pmod {4}, \end{array}\right. } \end{aligned}$$

where $A_4$ is a character of order 4. Note that the last equality follows by using the fact that if $\chi \psi \ne \varepsilon$ then $J(\phi ,\psi )=\frac{g(\chi )g(\psi )}{g(\chi \psi )}.$

Therefore, by (4.13) we have $_2\widetilde{G}_2(-1)_p=a_p(-1).$ Therefore, for $\lambda \ne 0,1$

$$\begin{aligned} _2\widetilde{G}_2(\lambda )_p=a_p(\lambda ). \end{aligned}$$

If $k\ge 4$ is even then Proposition 2.1 of [6] gives

4.14 $$\begin{aligned} {\textrm{Tr}_k}(\Gamma _0(4), p)&=-3-\sum _{\lambda =2}^{p-1}P_k(a_p(\lambda ),p), \end{aligned}$$

4.15 $$\begin{aligned} {\textrm{Tr}_k}(\Gamma _0(8), p)&=-4-\sum _{\lambda =2}^{p-1}P_k(a_p(\lambda ^2),p). \end{aligned}$$

Therefore, we conclude the results by replacing $a_p(\lambda )$ by $_2\widetilde{G}_2(\lambda )_p$ in the above two trace formulas. $\square$

### Proof of Theorem 2.2

The proof follows similarly as in the proof of Theorem 2.1. The only requirement is to show for $\lambda \ne 0,1$

$$\begin{aligned} _6\widetilde{G}_6(\lambda )_p=a_p(\lambda ). \end{aligned}$$

This is straightforward by revisiting (4.12), (4.13) and the definition of $_6\widetilde{G}_6(-1)_p.$

$$\square $$

## Data Availability

Data sharing is not applicable to this article as no datasets were generated or analysed during the current study.

## Footnotes

1 $\chi (0):=0$ for all multiplicative characters of $\mathbb {F}_q.$

2 $\omega (t)\equiv t\pmod {p}$ for all $t\in \mathbb {F}_p$ and $\omega (0):=0.$

3 For all multiplicative characters $\chi$ including the trivial character $\chi (0):=0.$
